# Supplementary material for: Huntington’s disease age at motor onset is modified by the tandem hexamer repeat in TCERG1
Source: NPJ Genom Med. 2022 Sep 5;7:53. doi: 10.1038/s41525-022-00317-w (PMC9445028; doi:10.1038/s41525-022-00317-w)
Supplement: Supplementary file 1 — Supplementary Information [file 41525_2022_317_MOESM1_ESM.docx]

**Supplementary Information for “Huntington’s disease age at motor onset is modified by the tandem hexamer repeat in *TCERG1*”**

**Supplementary Tables**

| Regression | $\mathcal{R}_{\text{thr}}$ | $N_{\text{early}}$ | $N_{\text{late}}$ | Sum | | Max | | Min | | Diff | |
| --- | --- | --- | --- | --- | --- | --- | --- | --- | --- | --- | --- |
|  |  |  |  | b | *p* | b | *p* | b | *p* | b | *p* |
| Linear | Not applicable | | | -2.74 | 5.0E-09 | -3.75 | 1.2E-03 | -2.85 | 1.8E-07 | 1.82 | 4.9E-04 |
| Logistic | 0 | 323 | 287 | -0.45 | 7.7E-08 | -0.66 | 1.9E-03 | -0.45 | 2.1E-06 | 0.27 | 1.9E-03 |
|  | 7 | 238 | 203 | -0.48 | 8.9E-07 | -0.86 | 1.3E-03 | -0.47 | 1.8E-05 | 0.28 | 5.9E-03 |
|  | 13 | 149 | 78 | -0.83 | 2.0E-09 | -66.68 | 7.1E-04 | -0.84 | 1.7E-08 | 0.65 | 5.0E-06 |
|  | 20 | 24 | 11 | -35.73 | 3.2E-05 | -51.33 | 7.9E-02 | -34.71 | 3.9E-05 | 1.37 | 5.1E-04 |

**Supplementary Table 1.** Significance of the association between TCERG1 exon 4 quasi-tandem repeat (QTR) and residual age at onset $\mathcal{R}$ for the various ways of coding the repeat. Blue and red colours highlight numbers passing ${10}^{-5}$ and $5\cdot{10}^{-8}$ *p*-value thresholds for significance, respectively.

$\mathcal{R}_{\text{thr}}$: residual age at onset threshold used for logistic regression (years)

$\mathbb{N}_{\text{early}}$: number of people with early onset used for logistic regression who have $\mathcal{R<-R}_{\text{thr}}$

$\mathbb{N}_{\text{late}}$: number of people with late onset used for logistic regression who have $\mathcal{R>R}_{\text{thr}}$

Max: QTR repeat length of the longest allele $N_{\text{max}}$

Min: QTR repeat length of the shortest allele $N_{\text{min}}$

Sum: sum of two QTR repeat lengths $N_{\text{sum}}=N_{\text{max}}+N_{\text{m}\text{in}}$

Diff: difference of two QTR lengths $N_{\text{diff}}=N_{\text{max}}-N_{\text{m}\text{in}}$

| Cohort | Type of regression analysis of REGISTRY | b | SE | CI | | *p* |
| --- | --- | --- | --- | --- | --- | --- |
| REGISTRY | Linear | -3.10 | 0.55 | -4.19 | -2.02 | 3.15E-08 |
|  | Selection | -0.98 | 0.19 | -1.36 | -0.62 | 2.30E-08 |
| Predict-HD | N/A | -1.26 | 0.56 | -2.37 | -0.14 | 0.027 |
| Combined | Linear | -2.74 | 0.46 | -3.65 | -1.84 | 5.02E-09 |
|  | Selection | -1.00 | 0.18 | -1.37 | -0.67 | 2.14E-09 |

**Supplementary Table 2.** Significance of the association between the sum of TCERG1 QTR lengths and residual age at onset in REGISTRY, Predict-HD and combined samples. Two types of regression analysis of the REGISTRY cohort are presented: linear regression analysis and regression with selection (see Supplementary Methods section below).

| Regression | $\mathcal{R}_{\text{thr}}$ | $N_{\text{early}}$ | $N_{\text{late}}$ | Sum | | Max | | Min | | Diff | |
| --- | --- | --- | --- | --- | --- | --- | --- | --- | --- | --- | --- |
|  |  |  |  | b | *p* | b | *p* | b | *p* | b | *p* |
| Linear | Not applicable | | | -2.75 | 6.5E-09 | -3.78 | 1.2E-03 | -2.85 | 2.4E-07 | 1.80 | 6.5E-04 |
| Logistic | 0 | 323 | 287 | -0.45 | 1.2E-07 | -0.64 | 2.5E-03 | -0.45 | 2.7E-06 | 0.27 | 2.0E-03 |
|  | 7 | 238 | 203 | -0.48 | 1.1E-06 | -0.86 | 1.3E-03 | -0.47 | 2.3E-05 | 0.28 | 7.4E-03 |
|  | 13 | 149 | 78 | -0.83 | 2.8E-09 | -66.68 | 7.1E-04 | -0.85 | 2.3E-08 | 0.65 | 7.3E-06 |
|  | 20 | 24 | 11 | -35.73 | 3.2E-05 | -51.33 | 7.9E-02 | -34.71 | 3.9E-05 | 1.37 | 5.1E-04 |

**Supplementary Table 3.** As **Supplementary Table 1**, but for short tandem repeat (STR).

| Baseline  model | Additional statistic | | | | |
| --- | --- | --- | --- | --- | --- |
|  | Sum | Max | Min | Diff | #3 repeats |
| Sum | X | 0.79 | 0.79 | 0.79 | 0.67 |
| Max | 1.07E-06 | X | 1.07E-06 | 1.07E-06 | 3.33E-05 |
| Min | 8.28E-03 | 8.28E-03 | X | 8.28E-03 | 0.71 |
| Diff | 2.61E-06 | 2.61E-06 | 2.61E-06 | X | 6.03E-03 |
| #3repeats | 1.29E-04 | 4.77E-03 | 5.49E-03 | 0.95 | X |

**Supplementary Table 4.** Significance (p-value) of improvement in fit to residual age at onset given by adding the “additional” QTR statistic to a model containing the “baseline” QTR statistic. See **Supplementary Table 1** for explanation of Sum, Max, Min, and Diff.

**Supplementary Data**

**eQTLGen-PPP2R2B**. List of significant eQTLGen eQTLs for PPP2R2B with corresponding p-value for association with age at onset in the GeM GWAS. “P-value” = eQTLGen eQTL p-value, “Z-score” = eQTLGen test statistic. Positive Z means that the “assessed” allele is associated with higher expression. “AAO_effect” is the increase (or decrease, if negative) in age at onset (years) associated in the GeM GWAS with one copy of the “assessed” allele. P(GeM) is the p-value for association with age at onset in the GeM GWAS.

**eQTLGen-TCERG1**. List of significant eQTLGen eQTLs for TCERG1 with corresponding p-value for association with age at onset in the GeM GWAS. “P-value” = eQTLGen eQTL p-value, “Z-score” = eQTLGen test statistic. Positive Z means that the “assessed” allele is associated with higher expression. “AAO_effect” is the increase (or decrease, if negative) in age at onset (years) associated in the GeM GWAS with one copy of the “assessed” allele. P(GeM) is the p-value for association with age at onset in the GeM GWAS.

**PsychENCODE-TCERG1**. List of significant psychENCODE eQTLs for TCERG1 with corresponding p-value for association with age at onset in the GeM GWAS. “eQTL_pval” = psychENCODE eQTL p-value, “eQTL_effect” = change in expression associated with each copy of allele A1. “AAO_effect” is the increase (or decrease, if negative) in age at onset (years) associated in the GeM GWAS with one copy of the “assessed” allele. P(GeM) is the p-value for association with age at onset in the GeM GWAS.

**PsychENCODE-PPP2R2B**. List of significant psychENCODE eQTLs for PPP2R2B with corresponding p-value for association with age at onset in the GeM GWAS. “eQTL_pval” = psychENCODE eQTL p-value, “eQTL_effect” = change in expression associated with each copy of allele A1. “AAO_effect” is the increase (or decrease, if negative) in age at onset (years) associated in the GeM GWAS with one copy of the “assessed” allele. P(GeM) is the p-value for association with age at onset in the GeM GWAS.

**Supplementary Figures**

MAERGGDGGESERFNPGELRMAQQQALRFRGPAPPPNAVMRGPPPLMRPPPPFGMMRGPPPPPRPPFGRP

PFDPNMPPMPPPGGIPPPMGPPHLQRPPFMPPPMSSMPPPPGMMFPPGMPPVTAPGTPALPPTEEIWVEN

KTPDGKVYYYNARTRESAWTKPDGVKVIQQSELTPMLAAQAQVQAQAQAQAQAQAQAQAQAQAQAQAQAQ

AQAQAQAQAQAQAQAQAQAQAQAQAQAQAQAQVQAQVQAQVQAQAVGASTPTTSSPAPAVSTSTSSSTPS

STTSTTTTATSVAQTVSTPTTQDQTPSSAVSVATPTVSVSTPAPTATPVQTVPQPHPQTLPPAVPHSVPQ

PTTAIPAFPPVMVPPFRVPLPGMPIPLPGVAMMQIVSCPYVKTVATTKTGVLPGMAPPIVPMIHPQVAIA

ASPATLAGATAVSEWTEYKTADGKTYYYNNRTLESTWEKPQELKEKEKLEEKIKEPIKEPSEEPLPMETE

EEDPKEEPIKEIKEEPKEEEMTEEEKAAQKAKPVATAPIPGTPWCVVWTGDERVFFYNPTTRLSMWDRPD

DLIGRADVDKIIQEPPHKKGMEELKKLRHPTPTMLSIQKWQFSMSAIKEEQELMEEINEDEPVKAKKRKR

MSKKSFMWIARASLFRRDDNKDIDSEKEAAMEAEIKAARERAIVPLEARMKQFKDMLLERGVSAFSTWEK

ELHKIVFDPRYLLLNPKERKQVFDQYVKTRAEEERREKKNKIMQAKEDFKKMMEEAKFNPRATFSEFAAK

HAKDSRFKAIEKMKDREALFNEFVAAARKKEKEDSKTRGEKIKSDFFELLSNHHLDSQSRWSKVKDKVES

DPRYKAVDSSSMREDLFKQYIEKIAKNLDSEKEKELERQARIEASLREREREVQKARSEQTKEIDREREQ

HKREEAIQNFKALLSDMVRSSDVSWSDTRRTLRKDHRWESGSLLEREEKEKLFNEHIEALTKKKREHFRQ

LLDETSAITLTSTWKEVKKIIKEDPRCIKFSSSDRKKQREFEEYIRDKYITAKADFRTLLKETKFITYRS

KKLIQESDQHLKDVEKILQNDKRYLVLDCVPEERRKLIVAYVDDLDRRGPPPPPTASEPTRRSTK

**Supplementary Figure 1.** Transcription elongation regulator 1 isoform 3 [Homo sapiens]. NCBI Reference Sequence: NP_001369477.1


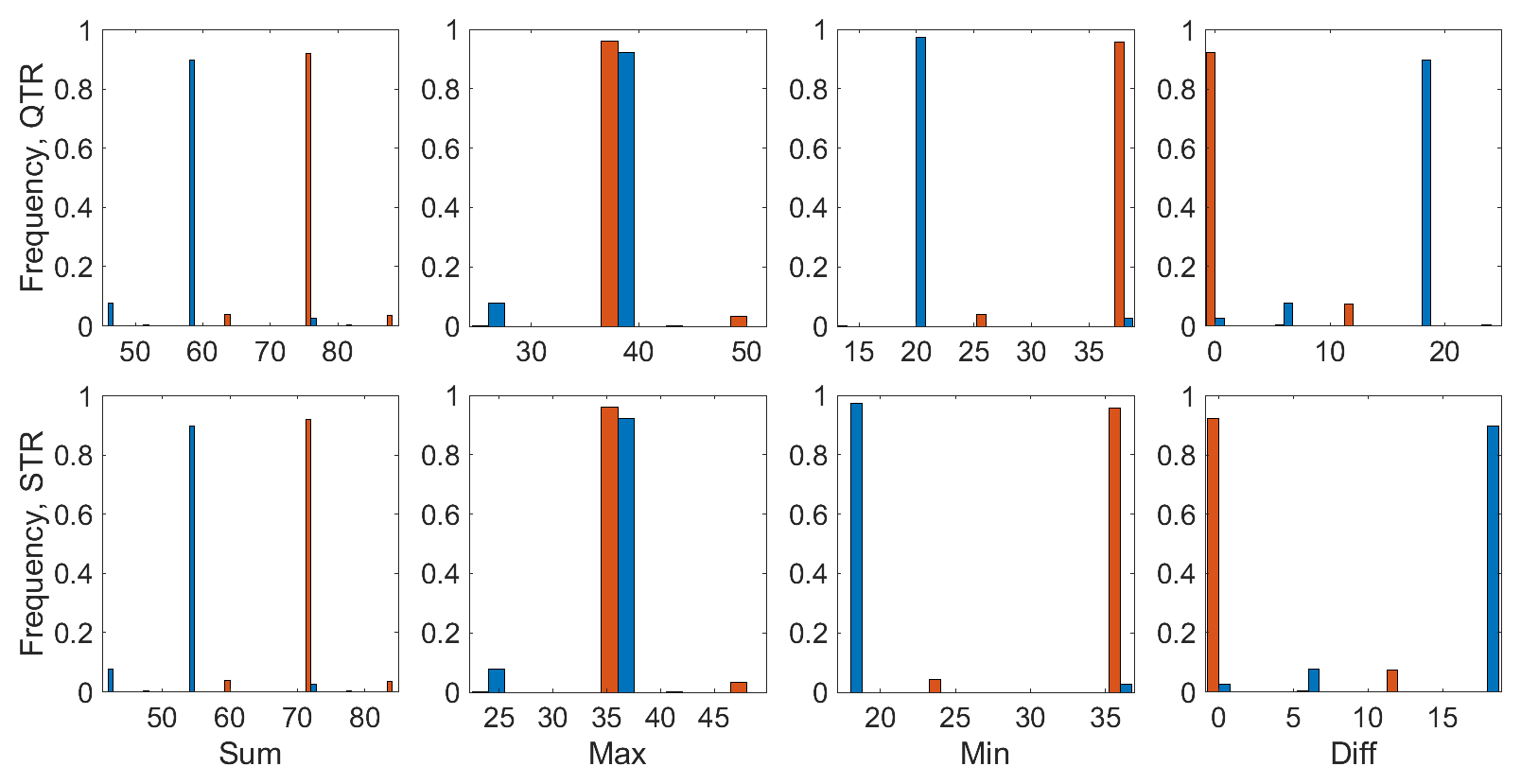


**Supplementary Figure 2.** Plots of the distribution of QTR (top) and STR (bottom) lengths in individuals with 0 (red) and 1 (blue) minor alleles at rs79727797 for the 468 individuals with both SNV and sequencing data. See **Supplementary Table 1** for explanation of Sum, Max, Min, and Diff.

**
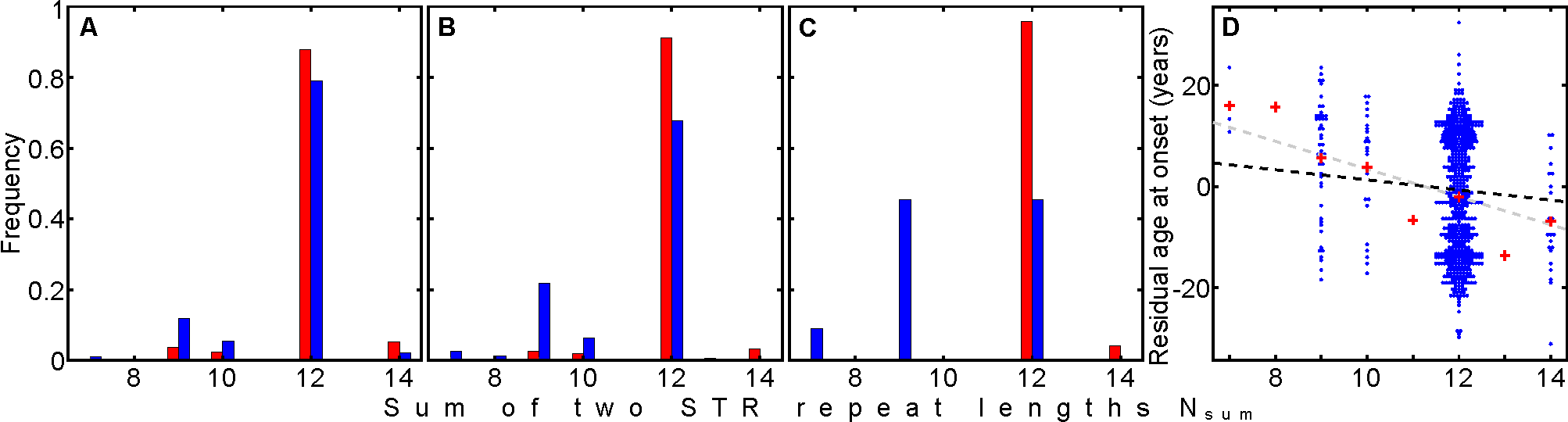
**

**Supplementary Figure 3.** The relationship between hexanucleotide short tandem repeat (STR) length and residual age at onset of HD. **a-c** Histograms showing distribution of the sum of two STR repeat lengths N_sum_ = N_min_ + N_max_ for the groups with early (red, R<-R_thr_) and late (blue, R>R_thr_) onsets. The panels **a**, **b**, and **c** correspond to the residual age at onset threshold R_thr_ of 0, 13, and 20 years, respectively. **d** Association of the sum of two STR repeat lengths N_sum_ with the residual age at onset for the entire HD cohort. Red pluses indicate mean residual age at onset for every sum of STR repeat lengths. Grey and black dashed lines are plotted using coefficients of the linear regression analysis and regression with selection.


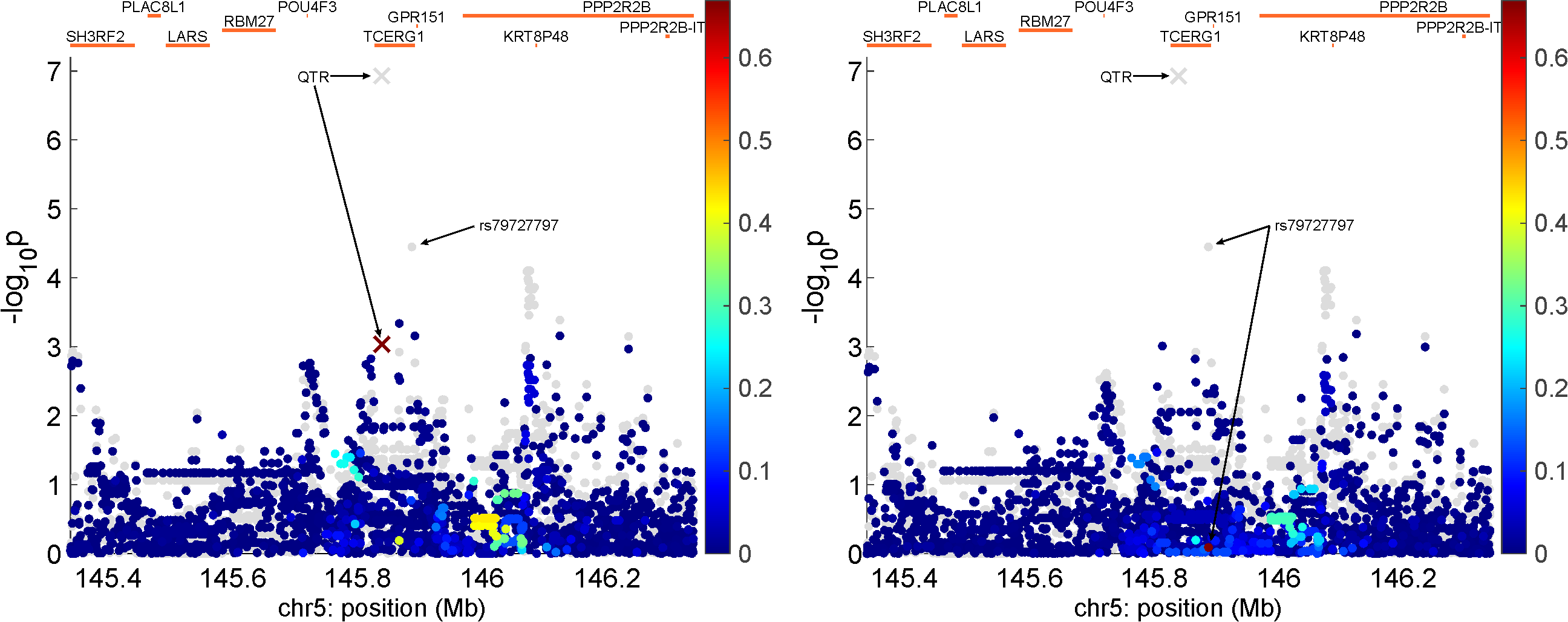


**Supplementary Figure 4.** Manhattan plots of residual age at onset association conditioning on rs79727797 (left panel) and QTR (right panel) for 468 HD individuals with both sequencing and GWAS data. The bar on the right of the plots indicates the strength of linkage disequilibrium (r2) between each SNP/QTR and the variant being conditioned on. The grey dots mark p-values prior to conditioning. The variant being conditioned on necessarily disappears from the plot.


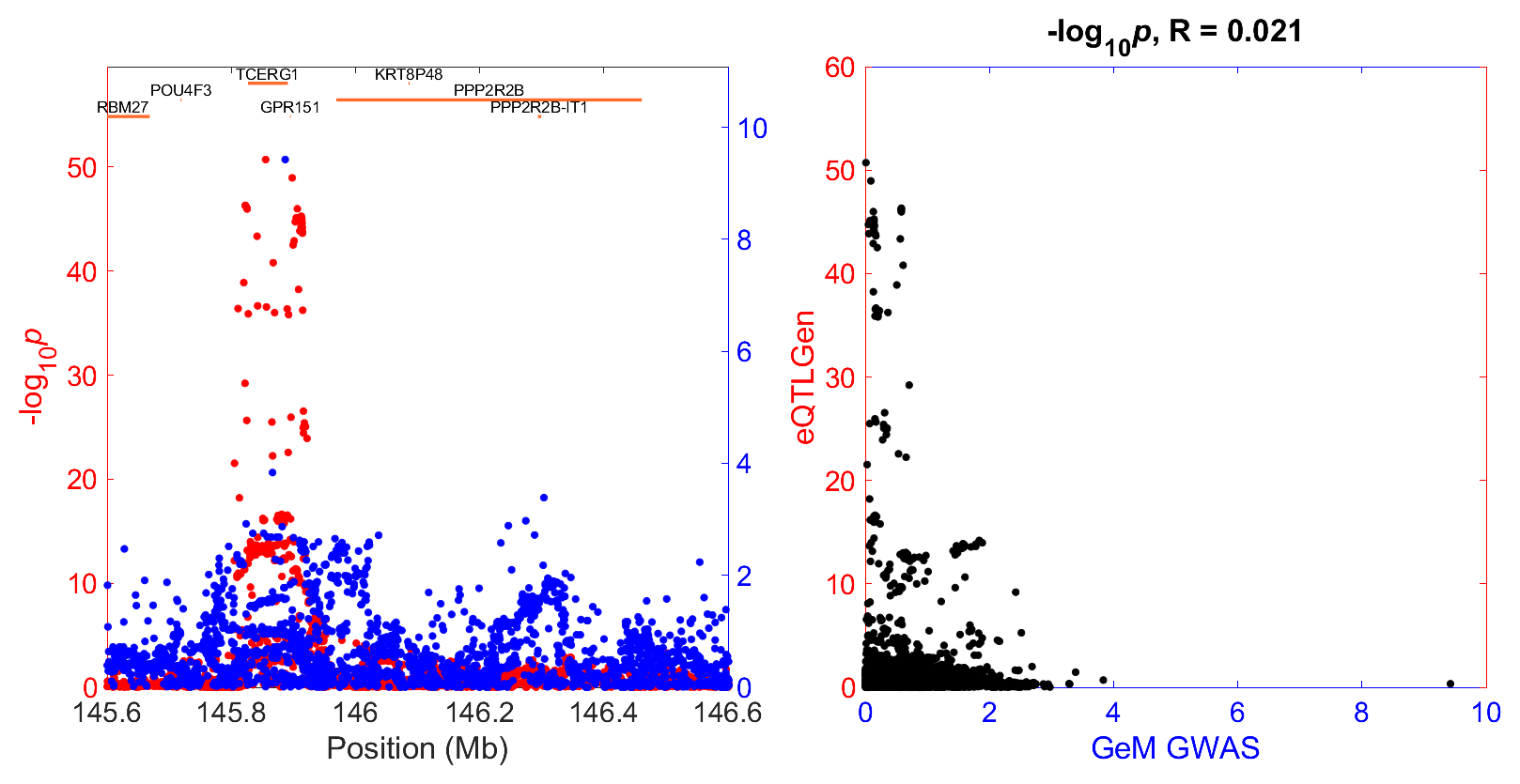


**Supplementary Figure 5**. Plots of TCERG1 eQTL -log p-value from eQTLGen (red) and GeM GWAS -log p-value (blue) vs chromosome position (left panel) and each other (right panel)


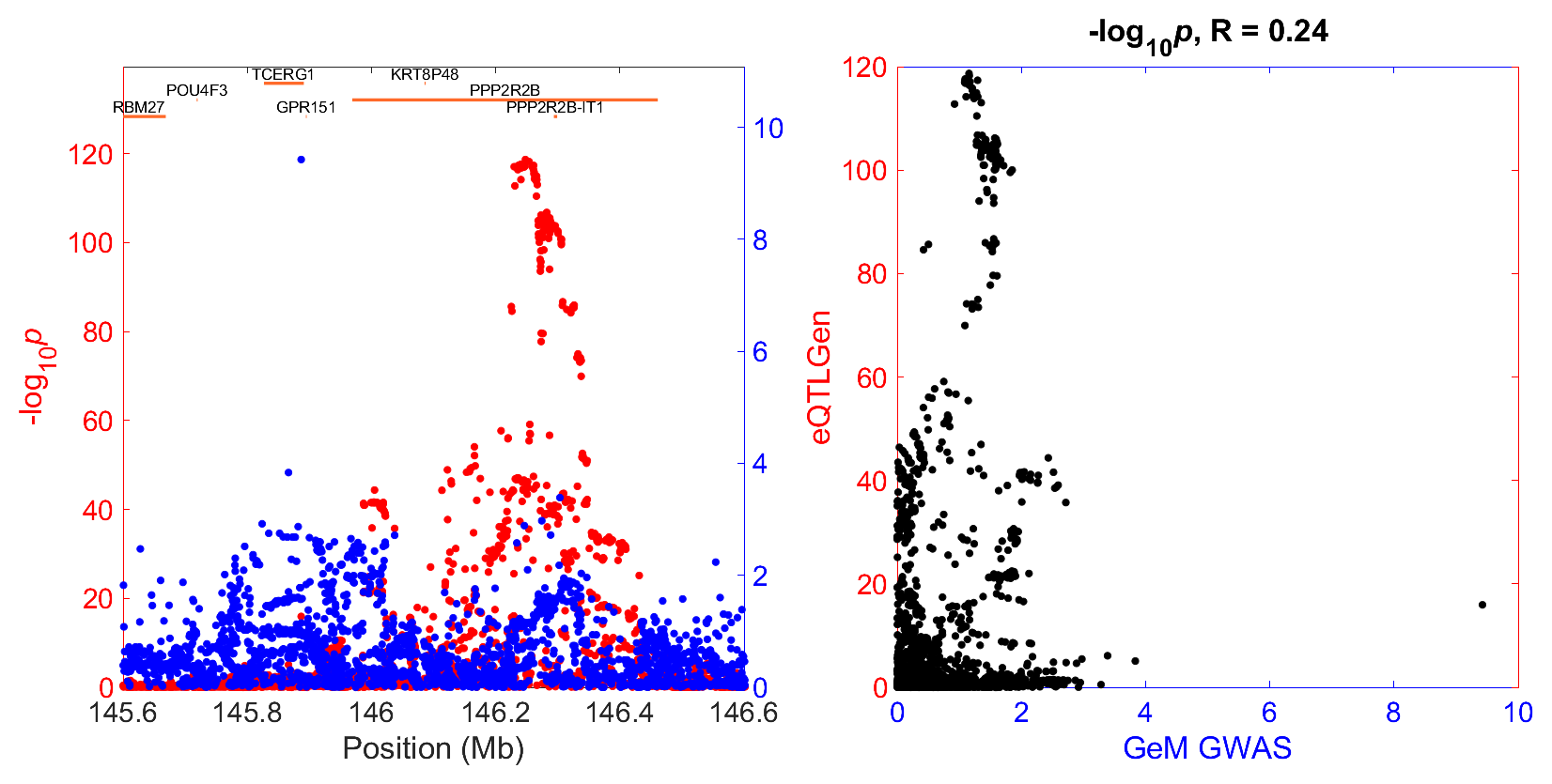


**Supplementary Figure 6**. Plots of PPP2R2B eQTL -log p-value from eQTLGen (red) and GeM GWAS -log p-value (blue) vs chromosome position (left panel) and each other (right panel)


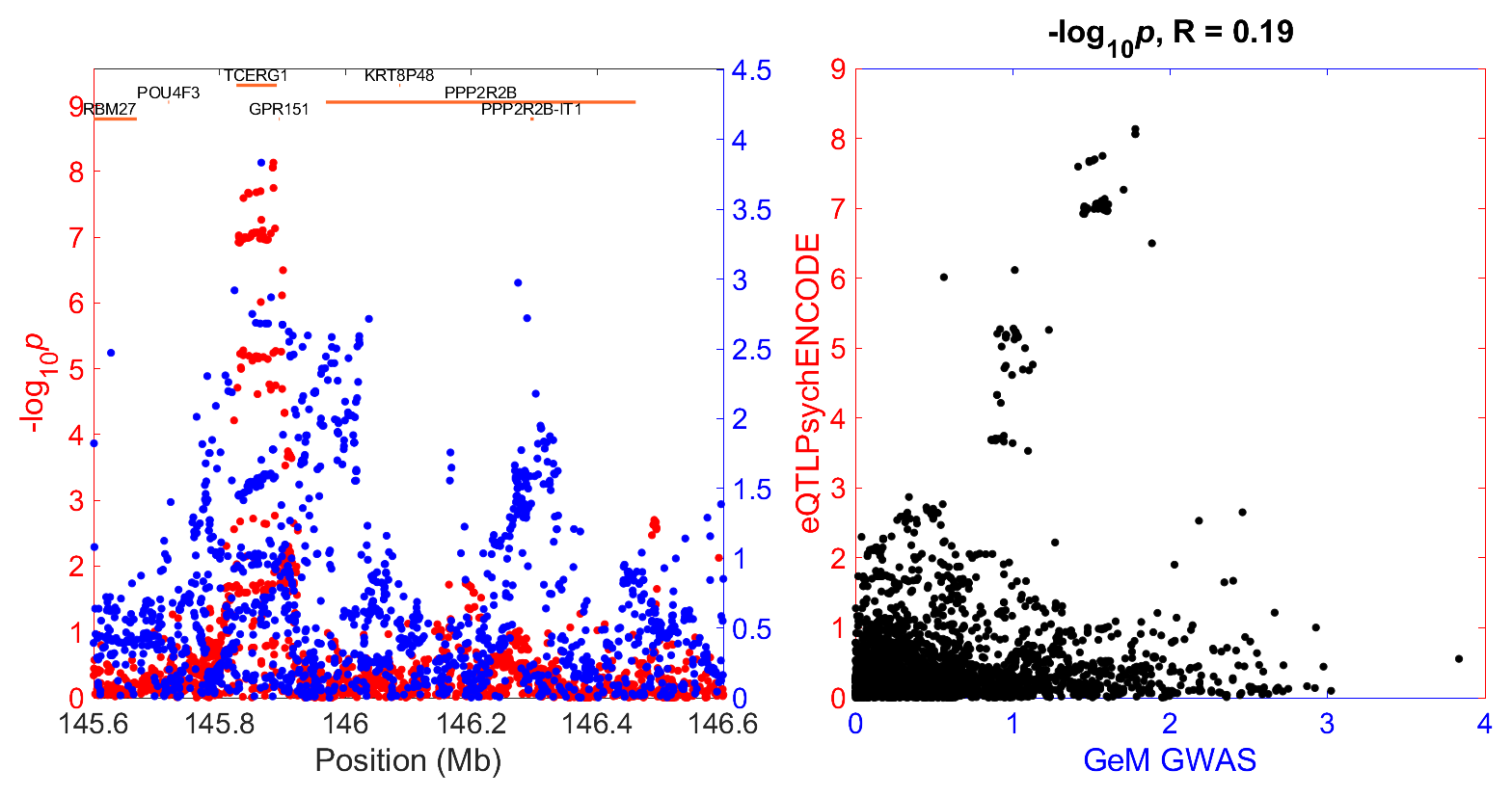


**Supplementary Figure 7**. Plots of TCERG1 eQTL -log p-value from PsychENCODE (red) and GeM GWAS -log p-value (blue) vs chromosome position (left panel) and each other (right panel)


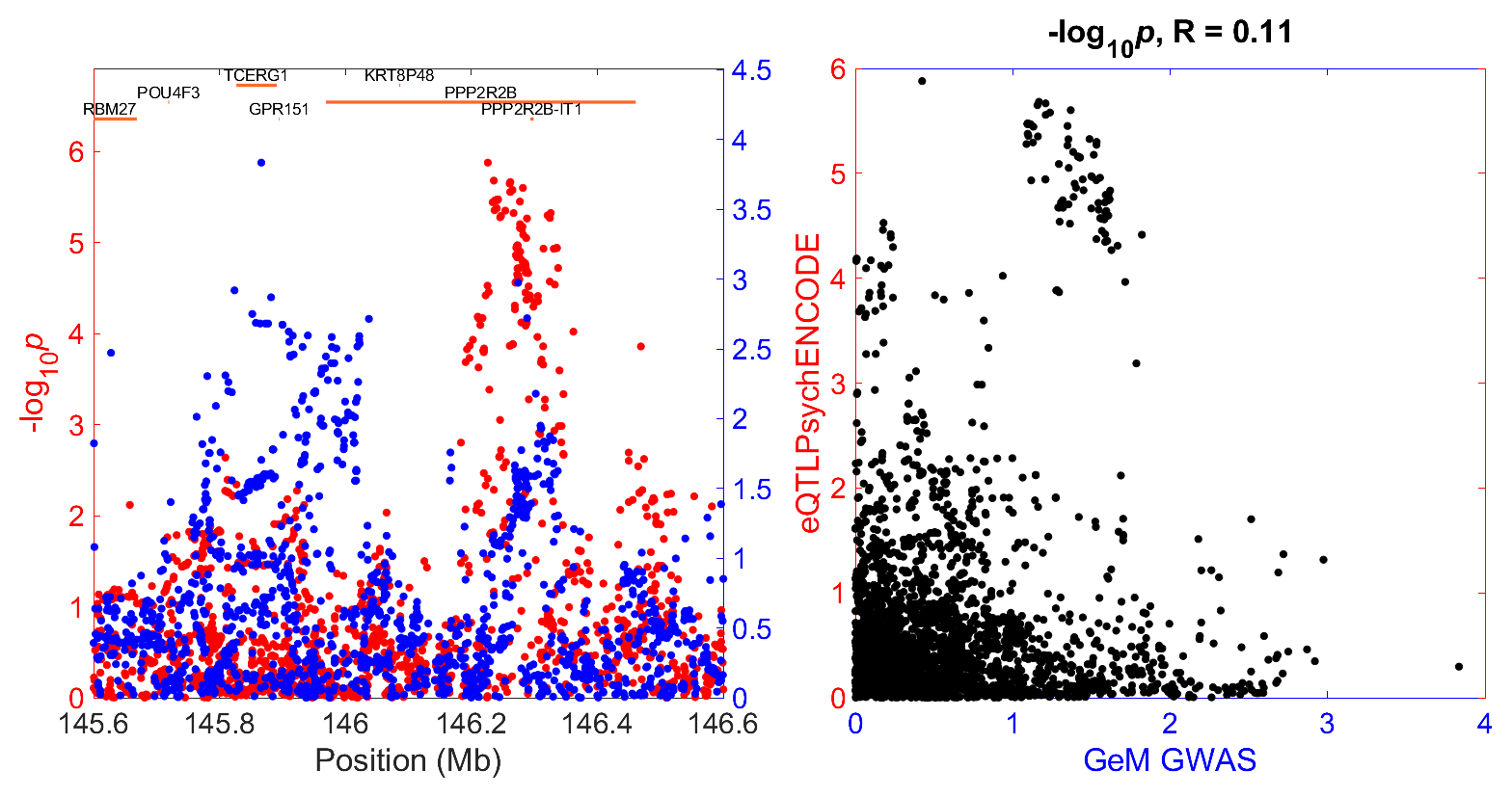


**Supplementary Figure 8**. Plots of PPP2R2B eQTL -log p-value from PsychENCODE (red) and GeM GWAS -log p-value (blue) vs chromosome position (left panel) and each other (right panel)


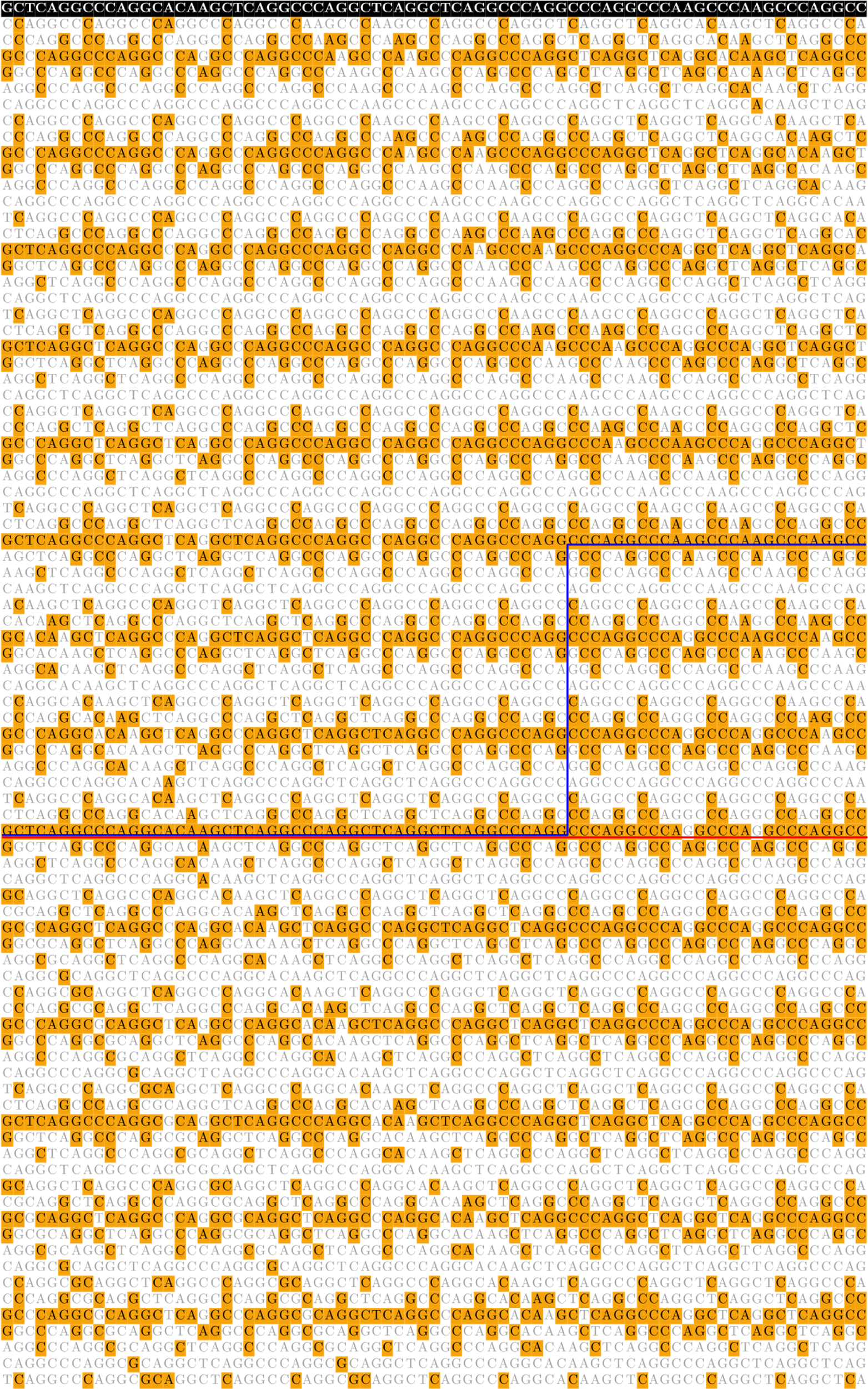


**Supplementary Figure 9**. Illustration of the match matrix. The top line with white letters on a black ground specifies the nucleotide sequence of a read. Other lines represent nucleotide sequences of the reference genome shifted by one nucleotide with respect to the previous (upper) line. Matched nucleotides are highlighted with a yellow colour which creates the match matrix (yellow = True, white = False). Two read alignments are shown. The naive way to align a read with two mismatched nucleotides is shown as a red straight line. The path with one deletion, which takes into account the highly mutative nature of STRs/QTRs, is shown with a blue line.


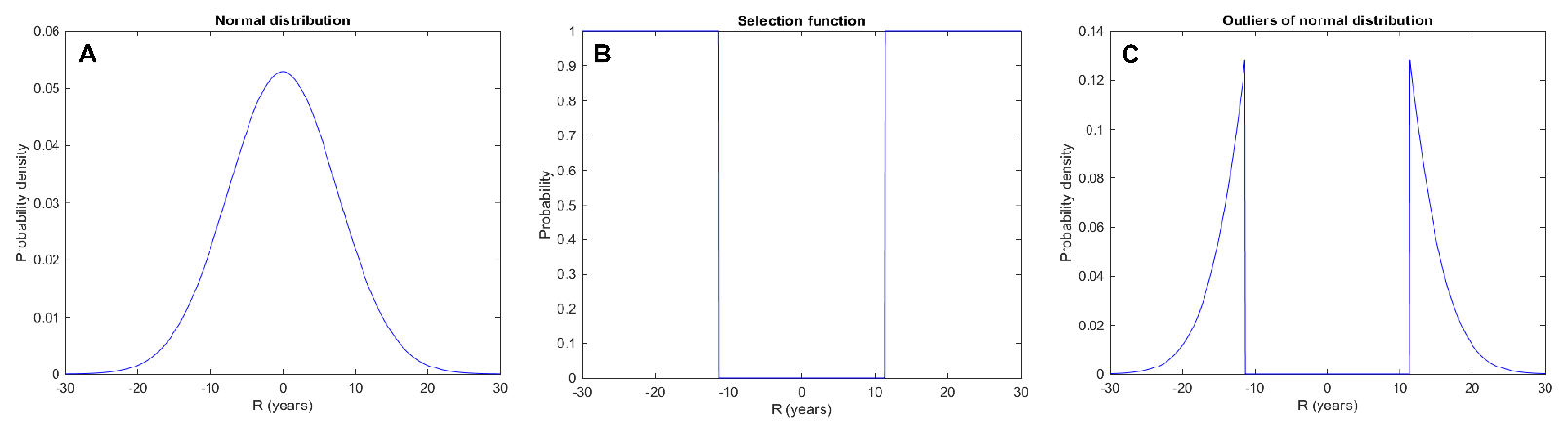


**Supplementary Figure 10**. **a** Expected probability density of the initial HD population (normal distribution); **b** Selection function with infinitely small $\Delta$; **c**Expected probability density of the small HD sub-group with largest absolute value of the residual age at onset $\left| R \right|$.


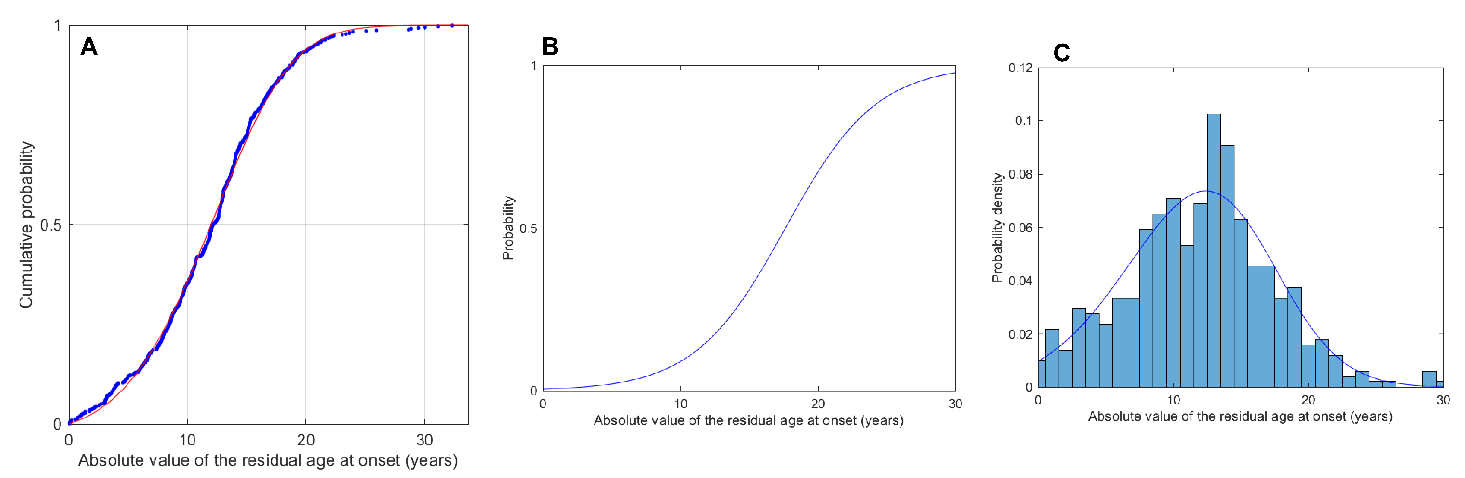


**Supplementary Figure 11**. **a** The observed (blue dots) and expected (red line) cumulative probabilities; **b** The selection function with optimal parameters; **c**The observed (bars) and expected (line) probability densities.

**Members of Consortia**

**REGISTRY Investigators of the European Huntington’s disease network**

**REGISTRY Steering committee:** Anne-Catherine Bachoud-Lévi, Anna Rita Bentivoglio, Ida Biunno, Raphael M Bonelli, Jean-Marc Burgunder, Stephen B Dunnett, Joaquim J Ferreira, Olivia J. Handley, Arvid Heiberg, Torsten Illmann, G Bernhard Landwehrmeyer, Jamie Levey, Maria A. Ramos-Arroyo, Jørgen E Nielsen, Susana Pro Koivisto, Markku Päivärinta, Raymund A.C. Roos, Ana Rojo Sebastián, Sarah J Tabrizi, Wim Vandenberghe, Christine Verellen-Dumoulin, Tereza Uhrova, Jan Wahlström, Jacek Zaremba

**Language coordinators:** Verena Baake, Katrin Barth, Monica Bascuñana Garde, Sabrina Betz, Reineke Bos, Jenny Callaghan, Adrien Come, Leonor Correia Guedes, Daniel Ecker, Ana Maria Finisterra, Ruth Fullam, Mette Gilling, Lena Gustafsson, Olivia J. Handley, Carina Hvalstedt, Christine Held, Kerstin Koppers, Claudia Lamanna, Matilde Laurà, Asunción Martínez Descals, Saül Martinez-Horta, Tiago Mestre, Sara Minster, Daniela Monza, Lisanne Mütze, Martin Oehmen, Michael Orth, Hélène Padieu, Laurent Paterski, Nadia Peppa, Susana Pro Koivisto, Martina Di Renzo, Amandine Rialland, Niini Røren, Pavla Šašinková, Erika Timewell, Jenny Townhill, Patricia Trigo Cubillo, Wildson Vieira da Silva, Marleen R van Walsem, Carina Whalstedt, Marie-Noelle Witjes-Ané , Grzegorz Witkowski, Abigail Wright, Daniel Zielonka, Eugeniusz Zielonka, Paola Zinzi

**AUSTRIA**

**Graz (Medizinische Universitäts Graz, Psychiatrie):** Raphael M Bonelli, Sabine Lilek, Karen Hecht, Brigitte Herranhof, Anna Holl (formerly Hödl), Hans-Peter Kapfhammer, Michael Koppitz, Markus Magnet, Nicole Müller, Daniela Otti, Annamaria Painold, Karin Reisinger, Monika Scheibl, Helmut Schöggl, Jasmin Ullah

**Innsbruck (Universitätsklinik Innsbruck, Neurologie):** Eva-Maria Braunwarth, Florian Brugger, Lisa Buratti, Eva-Maria Hametner, Caroline Hepperger, Christiane Holas, Anna Hotter, Anna Hussl, Christoph Müller, Werner Poewe, Klaus Seppi, Fabienne Sprenger, Gregor Wenning

**BELGIUM**

**Bierbeek:** Andrea Boogaerts, Godelinde Calmeyn, Isabelle Delvaux, Dirk Liessens, Nele Somers

**Charleroi (Institut de Pathologie et de Génétique (IPG)):** Michel Dupuit, Cécile Minet, Dominique van Paemel, Pascale Ribaï, Christine Verellen-Dumoulin

**Leuven:** (Universitair Ziekenhuis Gasthuisberg,): Andrea Boogaerts, Wim Vandenberghe, Dimphna van Reijen

**CZECH REPUBLIC**

**Prague (Extrapyramidové centrum, Neurologická klinika, 1. LF UK a VFN):** Jiří Klempíř, Veronika Majerová, Jan Roth, Irena Stárková

**DENMARK**

**Copenhagen (Neurogenetics Clinic, Danish Dementia Research Centre, Rigshospitalet, University of Copenhagen):** Lena E. Hjermind, Oda Jacobsen, Jørgen E Nielsen, Ida Unmack Larsen, Tua Vinther-Jensen

**FINLAND**

**Turku-Suvituuli (Rehabilitation Centre Suvituuli):** Heli Hiivola, Hannele Hyppönen, Kirsti Martikainen, Katri Tuuha

**FRANCE**

**Angers (Centre de référence des maladies neurogénétique- CHU d’Angers):** Philippe Allain, Dominique Bonneau, Marie Bost, Bénédicte Gohier, Marie-Anne Guérid, Audrey Olivier, Adriana Prundean, Clarisse Scherer-Gagou, Christophe Verny

**Bordeaux (Hôpital CHU Pellegrin):** Blandine Babiloni, Sabrina Debruxelles, Charlotte Duché, Cyril Goizet, Laetitia Jameau, Danielle Lafoucrière, Umberto Spampinato

**Lille-Amiens:**

**Lille (CHRU Roger Salengro) :** Rekha Barthélémy, Christelle De Bruycker, Maryline Cabaret, Anne-Sophie Carette, Eric Decorte  Luc Defebvre, Marie Delliaux, Arnaud Delval, Alain Destee, Kathy Dujardin, Marie-Hélène Lemaire, Sylvie Manouvrier, Mireille Peter, Lucie Plomhouse, Bernard Sablonnière, Clémence Simonin, Stéphanie Thibault-Tanchou, Isabelle Vuillaume

**Amiens (CHU Nord) :** Marcellin Bellonet, Hassan Berrissoul, Stéphanie Blin, Françoise Courtin, Cécile Duru, Véronique Fasquel, Olivier Godefroy, Pierre Krystkowiak, Béatrice Mantaux, Martine Roussel, Sandrine Wannepain

**Marseille (Hôpital La Timone) :** Jean-Philippe Azulay, Marie Delfini, Alexandre Eusebio, Frédérique Fluchere, Laura Mundler

**Strasbourg (Hôpital Civil) :** Mathieu Anheim, Celine Julié, Ouhaid Lagha Boukbiza, Nadine Longato, Gabrielle Rudolf, Christine Tranchant, Marie-Agathe Zimmermann

**GERMANY**

**Aachen (Universitätsklinikum Aachen, Neurologische Klinik):** Christoph Michael Kosinski, Eva Milkereit, Daniela Probst, Kathrin Reetz, Christian Sass, Johannes Schiefer, Christiane Schlangen, Cornelius J. Werner

**Berlin (Klinik und Poliklinik für Neurologie - Charité - Universitätsmedizin Berlin):** Harald Gelderblom, Josef Priller, Harald Prüß, Eike Jakob Spruth

**Bochum (Huntington-Zentrum (NRW) Bochum im St. Josef-Hospital):** Gisa Ellrichmann, Lennard Herrmann, Rainer Hoffmann, Barbara Kaminski, Peter Kotz, Christian Prehn, Carsten Saft

**Dinslaken (Reha Zentrum in Dinslaken im Gesundheitszentrums Lang):** Herwig Lange, Robert Maiwald

**Dresden (Universitätsklinikum Carl Gustav Carus an der Technischen Universität Dresden, Klinik und Poliklinik für Neurologie):** Matthias Löhle, Antonia Maass, Simone Schmidt, Cecile Bosredon, Alexander Storch, Annett Wolz, Martin Wolz

**Freiburg (Universitätsklinik Freiburg, Neurologie):** Philipp Capetian, Johann Lambeck, Birgit Zucker

**Hamburg (Universitätsklinikum Hamburg-Eppendorf, Klinik und Poliklinik für Neurologie):** Kai Boelmans, Christos Ganos, Walburgis Heinicke, Ute Hidding, Jan Lewerenz, Alexander Münchau, Michael Orth, Jenny Schmalfeld, Lars Stubbe, Simone Zittel

**Hannover (Neurologische Klinik mit Klinischer Neurophysiologie, Medizinische Hochschule Hannover):** Gabriele Diercks, Dirk Dressler, Heike Gorzolla, Christoph Schrader, Pawel Tacik

**Itzehoe (Schwerpunktpraxis Huntington, Neurologie und Psychiatrie):** Michael Ribbat

**Marburg KPP (Klinik für Psychiatrie und Psychotherapie Marburg-Süd):** Bernhard Longinus

**Marburg Uni (Universität Marburg, Neurologie):** Katrin Bürk, Jens Carsten Möller, Ida Rissling

**München (Huntington-Ambulanz im Neuro-Kopfzentrum - Klinikum rechts der Isar der Neurologischen Klinik und Poliklinik der Technischen Universität München):** Mark Mühlau, Alexander Peinemann, Michael Städtler, Adolf Weindl, Juliane Winkelmann, Cornelia Ziegler

**Münster (Universitätsklinikum Münster, Klinik und Poliklinik für Neurologie):** Natalie Bechtel, Heike Beckmann, Stefan Bohlen, Eva Hölzner, Herwig Lange, Ralf Reilmann, Stefanie Rohm, Silke Rumpf , Sigrun Schepers, Natalia Weber

**Taufkirchen (Isar-Amper-Klinikum - Klinik Taufkirchen (Vils)):** Matthias Dose, Gabriele Leythäuser, Ralf Marquard, Tina Raab, Alexandra Wiedemann

**Ulm (Universitätsklinikum Ulm, Neurologie):** Katrin Barth, Andrea Buck, Julia Connemann, Daniel Ecker, Carolin Geitner, Christine Held, Andrea Kesse, Bernhard Landwehrmeyer, Christina Lang, Jan Lewerenz, Franziska Lezius, Solveig Nepper, Anke Niess, Michael Orth, Ariane Schneider, Daniela Schwenk, Sigurd Süßmuth, Sonja Trautmann, Patrick Weydt

**ITALY**

**Bari Clinica Neurologica - Neurophysiopatology of Pain Unit UNIVERSITA' DI BARI):** Claudia Cormio, Vittorio Sciruicchio, Claudia Serpino, Marina de Tommaso

**Bologna (DIBINEM - Alma Mater Studiorum - Università di Bologna; IRCCS Istituto delle Scienze Neurologiche di Bologna):** Sabina Capellari, Pietro Cortelli, Roberto Galassi, Rizzo Giovanni, Roberto Poda, Cesa Scaglione

**Florence (Dipartimento di Scienze Neurologiche e Psichiatriche Universita' degli Studi di Firenze-Azienda Ospedaliera Universitaria Careggi):** Elisabetta Bertini, Elena Ghelli, Andrea Ginestroni, Francesca Massaro, Claudia Mechi, Marco Paganini, Silvia Piacentini, Silvia Pradella, Anna Maria Romoli, Sandro Sorbi

**Genoa (Dipartimento di Neuroscienze, Riabilitazione, Oftalmologia, Genetica e Scienze Materno-Infantili, Università di Genova):** Giovanni Abbruzzese, Monica Bandettini di Poggio, Giovanna Ferrandes, Paola Mandich, Roberta Marchese

**Milan (Fondazione IRCCS Istituto Neurologico Carlo Besta):**
Alberto Albanese, Daniela Di Bella, Anna Castaldo, Stefano Di Donato, Cinzia Gellera, Silvia Genitrini, Caterina Mariotti, Daniela Monza, Lorenzo Nanetti, Dominga Paridi, Paola Soliveri, Chiara Tomasello

**Naples (Dipartimento di Neuroscienze, Scienze Riproduttive e Odontostomatologiche, Università Federico II):** Giuseppe De Michele, Luigi Di Maio, Marco Massarelli, Silvio Peluso, Alessandro Roca, Cinzia Valeria Russo, Elena Salvatore, Pierpaolo Sorrentino

**Pozzilli (IS) (Centro di Neurogenetica e Malattie Rare - IRCCS Neuromed):** Enrico Amico, Mariagrazia Favellato, Annamaria Griguoli, Irene Mazzante, Martina Petrollini, Ferdinando Squitieri and **Rome (Lega Italiana Ricerca Huntington e malattie correlate - onlus / www.LIRH.it):** Barbara D'Alessio, Chiara Esposito

**Rome (Istituto di Farmacologia Traslazionale & Istituto di Scienze e Tecnologie della Cognizione /CNR, Istituto di Neurologia Università Cattolica del Sacro Cuore):** Anna Rita Bentivoglio, Marina Frontali, Arianna Guidubaldi, Tamara Ialongo, Gioia Jacopini, Carla Piano, Silvia Romano, Francesco Soleti, Maria Spadaro, Paola Zinzi

**NETHERLANDS**

**Enschede (Medisch Spectrum Twente):** Monique S.E. van Hout, Marloes E. Verhoeven, Jeroen P.P. van Vugt, A. Marit de Weert

**Groningen (Polikliniek Neurologie**): J.J.W. Bolwijn, M. Dekker, B. Kremer, K.L. Leenders, J.C.H. van Oostrom

**Leiden (Leiden University Medical Centre (LUMC**)): Simon J. A. van den Bogaard, Reineke Bos, Eve M. Dumas, Ellen P. ‘t Hart, Raymund A.C. Roos

**Nijmegen (Universitair Medisch Centrum St. Radboud, Neurology**): Berry Kremer, C.C.P. Verstappen

**NORWAY**

**Oslo University Hospital (Rikshospitalet, Dept. of Medical Genetics and Dept. of Neurology):** Olaf Aaserud, Jan Frich C., Arvid Heiberg, Marleen R. van Walsem, Ragnhild Wehus

**Oslo University Hospital (Ulleval**, **Dept. of Medical Genetics and Dept.of Neurorehabilitation)**: Kathrine Bjørgo, Madeleine Fannemel, Per F. Gørvell, Eirin Lorentzen, Susana Pro Koivisto, Lars Retterstøl, Bodil Stokke

**Trondheim (St. Olavs Hospital):** Inga Bjørnevoll, Sigrid Botne Sando

**POLAND**

**Gdansk (St. Adalbert Hospital, Gdansk, Medical University of Gdansk, Neurological and Psychiatric Nursing Dpt.):** Artur Dziadkiewicz, Malgorzata Nowak, Piotr Robowski, Emilia Sitek, Jaroslaw Slawek, Witold Soltan, Michal Szinwelski

**Katowice (Medical University of Silesia, Katowice):** Magdalena Blaszcyk, Magdalena Boczarska-Jedynak, Ewelina Ciach-Wysocka, Agnieszka Gorzkowska, Barbara Jasinska-Myga, Gabriela Kłodowska–Duda, Gregorz Opala, Daniel Stompel

**Krakow (Krakowska Akademia Neurologii):** Krzysztof Banaszkiewicz, Dorota Boćwińska, Kamila Bojakowska-Jaremek, Małgorzata Dec, Malgorzata Krawczyk, Monika Rudzińska, Elżbieta Szczygieł, Andrzej Szczudlik, Anna Wasielewska, Magdalena Wójcik

**Poznan (Poznan University of Medical Sciences, Poland):** Anna Bryl, Anna Ciesielska, Aneta Klimberg, Jerzy Marcinkowski, Husam Samara, Justyna Sempołowicz, Daniel Zielonka

**Warsaw-MU (Medical University of Warsaw, Neurology):** Anna Gogol (formerly Kalbarczyk), Piotr Janik, Hubert Kwiecinski, Zygmunt Jamrozik

**Warsaw-IPiN (Institute of Psychiatry and Neurology Dep. of Genetics, First Dep. of Neurology):** Jakub Antczak, Katarzyna Jachinska, Wioletta Krysa, Maryla Rakowicz, Przemyslaw Richter, Rafal Rola, Danuta Ryglewicz, Halina Sienkiewicz-Jarosz, Iwona Stępniak, Anna Sułek, Grzegorz Witkowski, Jacek Zaremba, Elzbieta Zdzienicka, Karolina Zieora-Jakutowicz

**PORTUGAL**

**Coimbra (Hospital Universitário de Coimbra):** Cristina Januário, Filipa Júlio

**Lisbon (Clinical Pharmacology Unit, Instituto de Medicina Molecular, Faculty of Medicine, University of Lisbon):** Joaquim J Ferreira, Miguel Coelho, Leonor Correia Guedes, Tiago Mendes, Tiago Mestre, Anabela Valadas

**Porto (Hospital de São João, (Faculdade de Medicina da Universidade do Porto)):** Carlos Andrade, Miguel Gago, Carolina Garrett, Maria Rosália Guerra.

**SPAIN**

**Badajoz (Hospital Infanta Cristina):** Carmen Durán Herrera, Patrocinio Moreno Garcia

**Barcelona-Hospital Mútua de Terrassa:** Miquel Aguilar Barbera, Dolors Badenes Guia, Laura Casas Hernanz , Judit López Catena, Pilar Quiléz Ferrer, Ana Rojo Sebastián, Gemma Tome Carruesco

**Barcelona-Bellvitge (Hospital Universitari de Bellvitge):** Jordi Bas, Núria Busquets, Matilde Calopa

**Barcelona-Merced (Hospital Mare de Deu de La Merced):** Misericordia Floriach Robert, Celia Mareca Viladrich, Jesús Miguel Ruiz Idiago, Antonio Villa Riballo

**Burgos (Servicio de Neurología Hospital General Yagüe):** Esther Cubo, Cecilia Gil Polo, Natividad Mariscal Perez, Jessica Rivadeneyra

**Granada (Hospital Universitario San Cecilio, Neurología**): Francisco Barrero, Blas Morales

**Madrid-Clinico (Hospital Clínico Universitario San Carlos):** María Fenollar, Rocío García-Ramos García, Paloma Ortega, Clara Villanueva

**Madrid RYC (Hospital Ramón y Cajal, Neurología):** Javier Alegre, Mónica Bascuñana, Juan Garcia Caldentey, Marta Fatás Ventura, Guillermo García Ribas, Justo García de Yébenes, José Luis López-Sendón Moreno, Patricia Trigo Cubillo

**Madrid FJD (Madrid-Fundación Jiménez Díaz):** Javier Alegre, Fernando Alonso Frech, Justo García de Yébenes, Pedro J García Ruíz, Asunción Martínez-Descals, Rosa Guerrero, María José Saiz Artiga, Vicenta Sánchez

**Murcia (Hospital Universitario Virgen de la Arrixaca):** María Fuensanta Noguera Perea, Lorenza Fortuna, Salvadora Manzanares, Gema Reinante, María Martirio Antequera Torres, Laura Vivancos Moreau

**Oviedo (Hospital Central de Asturias):** Sonia González González, Luis Menéndez Guisasola, Carlos Salvador, Esther Suaréz San Martín

**Palma de Mallorca (Hospital Universitario Son Espases):** Inés Legarda Ramirez, Aranzazú Gorospe, Mónica Rodriguez Lopera, Penelope Navas Arques, María José Torres Rodríguez, Barbara Vives Pastor

**Pamplona (Complejo Hospitalario de Navarra):** Itziar Gaston, Maria Dolores Martinez-Jaurrieta, Maria A. Ramos-Arroyo

**Sevilla ("Hospital Virgen Macarena"):** Jose Manuel Garcia Moreno, Carolina Mendez Lucena, Fatima Damas Hermoso, Eva Pacheco Cortegana, José Chacón Peña, Luis Redondo

**Sevilla (Hospital Universitario Virgen del Rocío):** Fátima Carrillo, María Teresa Cáceres, Pablo Mir, María José Lama Suarez, Laura Vargas-González

**Valencia (Hospital la Fe):** Maria E. Bosca, Francisco Castera Brugada, Juan Andres Burguera, Anabel Campos Garcia, Carmen Peiró Vilaplana

**SWEDEN**

**Göteborg (Sahlgrenska University Hospital**): Peter Berglund, Radu Constantinescu, Gunnel Fredlund, Ulrika Høsterey-Ugander, Petra Linnsand, Liselotte Neleborn-Lingefjärd, Jan Wahlström, Magnus Wentzel

**Umeå (Umeå University Hospital**): Ghada Loutfi, Carina Olofsson, Eva-Lena Stattin, Laila Westman, Birgitta Wikström

**SWITZERLAND**

**Bern:** Jean-Marc Burgunder, Yanik Stebler **(Swiss HD Zentrum)**, Alain Kaelin, Irene Romero, Michael Schüpbach, Sabine Weber Zaugg **(Zentrum für Bewegungsstörungen, Neurologische Klinik und Poliklinik, Universität Bern)**

**Zürich (Department of Neurology, University Hospital Zürich):** Maria Hauer, Roman Gonzenbach, Hans H. Jung, Violeta Mihaylova, Jens Petersen

**UNITED KINGDOM**

**Aberdeen (NHS Grampian Clinical Genetics Centre & University of Aberdeen):** Roisin Jack, Kirsty Matheson, Zosia Miedzybrodzka, Daniela Rae, Sheila A Simpson, Fiona Summers, Alexandra Ure, Vivien Vaughan

**Birmingham (The Barberry Centre, Dept of Psychiatry**): Shahbana Akhtar, Jenny Crooks, Adrienne Curtis, Jenny de Souza (Keylock), John Piedad, Hugh Rickards, Jan Wright

**Bristol (North Bristol NHS Trust, Southmead hospital):** Elizabeth Coulthard, Louise Gethin, Beverley Hayward, Kasia Sieradzan, Abigail Wright

**Cambridge (Cambridge Centre for Brain Repair, Forvie Site):** Matthew Armstrong, Roger A. Barker, Deidre O’Keefe, Anna Di Pietro, Kate Fisher, Anna Goodman, Susan Hill, Ann Kershaw, Sarah Mason, Nicole Paterson, Lucy Raymond, Rachel Swain, Natalie Valle Guzman

**Cardiff (Schools of Medicine and Biosciences, Cardiff University**): Monica Busse, Cynthia Butcher, Jenny Callaghan, Stephen Dunnett, Catherine Clenaghan, Ruth Fullam, Olivia Handley, Sarah Hunt, Lesley Jones, Una Jones, Hanan Khalil, Sara Minster, Michael Owen, Kathleen Price, Anne Rosser, Jenny Townhill

**Edinburgh (Molecular Medicine Centre, Western General Hospital, Department of Clinical Genetics):** Maureen Edwards, Carrie Ho (Scottish Huntington´s Association), Teresa Hughes (Scottish Huntington´s Association), Marie McGill, Pauline Pearson, Mary Porteous, Paul Smith (Scottish Huntington´s Association)

**Fife (Scottish Huntington's Association Whyteman's Brae Hospital):** Peter Brockie, Jillian Foster, Nicola Johns, Sue McKenzie, Jean Rothery, Gareth Thomas, Shona Yates

**Gloucester (Department of Neurology Gloucestershire Royal Hospital):** Liz Burrows, Carol Chu, Amy Fletcher, Deena Gallantrae, Stephanie Hamer, Alison Harding, Stefan Klöppel, Alison Kraus, Fiona Laver, Monica Lewis, Mandy Longthorpe, Ivana Markova, Ashok Raman, Nicola Robertson, Mark Silva, Aileen Thomson, Sue Wild, Pam Yardumian

**Hull (Castle Hill Hospital):** Carol Chu, Carole Evans, Deena Gallentrae, Stephanie Hamer, Alison Kraus, Ivana Markova, Ashok Raman

**Leeds (Chapel Allerton Hospital, Department of Clinical Genetics):** Leeds (Chapel Allerton Hospital, Clinical Genetics): Carol Chu, Stephanie Hamer, Emma Hobson, Stuart Jamieson, Alison Kraus, Ivana Markova, Ashok Raman, Hannah Musgrave, Liz Rowett, Jean Toscano, Sue Wild, Pam Yardumian

**Leicester (Leicestershire Partnership Trust, Mill Lodge):** Colin Bourne, Jackie Clapton, Carole Clayton, Heather Dipple, Dawn Freire-Patino, Janet Grant, Diana Gross, Caroline Hallam, Julia Middleton, Ann Murch, Catherine Thompson

**Liverpool (Walton Centre for Neurology and Neurosurgery**)**:** Sundus Alusi, Rhys Davies, Kevin Foy, Emily Gerrans, Louise Pate

**London (Guy's Hospital):** Thomasin Andrews, Andrew Dougherty, Charlotte Golding, Fred Kavalier, Hana Laing, Alison Lashwood, Dene Robertson, Deborah Ruddy, Alastair Santhouse, Anna Whaite

**London (The National Hospital for Neurology and Neurosurgery**): Thomasin Andrews, Stefania Bruno, Karen Doherty, Charlotte Golding, Salman Haider, Davina Hensman, Nayana Lahiri, Monica Lewis, Marianne Novak, Aakta Patel, Nicola Robertson, Elisabeth Rosser, Sarah Tabrizi, Rachel Taylor, Thomas Warner, Edward Wild

**Manchester (Genetic Medicine, University of Manchester, Manchester Academic Health Sciences Centre and Central Manchester University Hospitals NHS Foundation Trust):** Natalie Arran, Judith Bek, Jenny Callaghan, David Craufurd, Ruth Fullam, Marianne Hare, Liz Howard, Susan Huson, Liz Johnson, Mary Jones, Helen Murphy, Emma Oughton, Lucy Partington-Jones, Dawn Rogers, Andrea Sollom, Julie Snowden, Cheryl Stopford, Jennifer Thompson, Iris Trender-Gerhard, Nichola Verstraelen (formerly Ritchie), Leann Westmoreland

**Oxford (Oxford University Hospitals NHS Trust, Dept. of Neurosciences, University of Oxford):** Richard Armstrong, Kathryn Dixon, Andrea H Nemeth, Gill Siuda, Ruth Valentine

**Plymouth (Plymouth Huntington Disease Service, Mount Gould Hospital):** David Harrison, Max Hughes, Andrew Parkinson, Beverley Soltysiak

**Sheffield (The Royal Hallamshire Hospital– Sheffield Children’s Hospital):** Oliver Bandmann, Alyson Bradbury, Paul Gill, Helen Fairtlough, Kay Fillingham, Isabella Foustanos, Mbombe Kazoka, Kirsty O’Donovan, Nadia Peppa, Cat Taylor, Katherine Tidswell, Oliver Quarrell

**EHDN’s associate site in Singapore: National Neuroscience Institute Singapore:** Jean-Marc Burgunder, Puay Ngoh Lau, Emmanul Pica, Louis Tan

**PREDICT-HD Investigators of the Huntington Study Group**

| **Leadership and Infrastructure** |  |
| --- | --- |
| **Principal Investigator** |  |
| Jane S. Paulsen | University of Iowa |
| **PREDICT Steering Committee Members** | |
| **2008-2014** 3^rd^ NIH grant |  |
| H. Jeremy Bockholt Thomas Brashers-Krug Phil Danzer  Megan Smith Jeffrey D. Long | University of Iowa University of Iowa University of Iowa University of Iowa  University of Iowa |
| Hans J. Johnson | University of Iowa |
| Amanda Miller | University of Iowa |
| Kelsey Montross | University of Iowa |
| Holly Westerveld | Brown University |
| **2004-2007** 2^nd^ NIH grant and CHDI |  |
| Elizabeth Aylward | University of Washington, Seattle |
| Kevin Biglan | University of Rochester |
| Robi Blumenstein | HighQ/CHDI |
| Elise Kayson | University of Rochester |
| Hans Johnson | University of Iowa |
| Bernhard Landwehrmeyer | University of Ulm, Germany |
| Blair Leavitt | UBC, Canada |
| Marcy MacDonald | MGH, Boston |
| Kevin Duff | University of Iowa |
| James Mills | University of Iowa |
| Christopher Ross | Johns Hopkins University |
| Ethan Singner | HighQ |
| Margaret Sutherland | NINDS |
| Dan Van Kammen | HighQ/CHDI |
| **1999-2003** 1^st^ NIH grant |  |
| Elizabeth Aylward | University of Washington, Seattle |
| Mark Guttman  Michael Hayden | University of Toronto, Canada  University of British Columbia, Canada |
| Elise Kayson | University of Rochester |
| Karl Kieburtz Douglas Langbehn  Martha Nance | University of Rochester University of Iowa  Hennepin Hospital, Minneapolis |
| David Oakes  Christopher Ross | University of Rochester  Johns Hopkins University |
| Aileen Shinaman | University of Rochester |
| Ira Shoulson | University of Rochester |
| Julie Stout | Indiana University, Bloomington |
| **2014-2017** (Leadership for Ancillary Grants) | |
| H. Jeremy Bockholt | University of Iowa |
| Hans Johnson | University of Iowa |

| Daniel Sewell | University of Iowa |
| --- | --- |
| Ying Zhang | Indiana University |
| Roland Zschiegner | University of Iowa |
| Kelsey Montross | University of Iowa |
| Deborah Harrington | UCSD |
| Jessica Turner | Georgia State University |
| **Core Sections** |  |
| **Biomarker Working Group** |  |
| Jane Paulsen | University of Iowa |
| Stefano DiDonato | Fondazione IRCCS Institute of Neurology |
| Ken Evans | Ontario Cancer Biomarker Network |
| Daniel Chelsky | Caprion |
| Rick Meyers | Boston University |
| Dean Jones | Emory University |
| Clemens Scherzer | MGH |
| Andrew Juhl | University of Iowa |
| Beth Borowsky | CHDI |
| Blair Leavitt | Centre for Molecular Medicine/Therapeutics, UBC |
| Wayne Mattson | E N Rodgers Mem Hospital & Bedford VAMC |
| Asa Petersen | Lund University |
| Sarah Tabrizi | National Hospital for Neurology Neurosurgery UK |
| David Weir | UBC |
| **Brain Donation Working Group** |  |
| Jane Paulsen | University of Iowa |
| Anne Leserman | University of Iowa |
| Carol Moscowitz | Columbia University |
| Jean Paul G. Vonsattel | Columbia University |
| Stacie Vik | University of Iowa |
| **Cognitive Coordination Centers:** |  |
| Deborah Harrington | UCSD |
| Tamara Hershey | WUSL |
| Leigh Beglinger, Kevin Duff, Megan Smith | University of Iowa |
| Holly Westervelt | Brown University |
| Geoff Tremont | Brown University |
| Mary Gover | Brown University |
| Melanie Faust | Rhode Island Hospital |
| Greg Elias | Rhode Island Hospital |
| Jennifer Davis | Brown University |
| Susan Bonner | Rhode Island Hospital |
| Rachel Bernier | Rhode Island Hospital |
| Julie C. Stout | Indiana Univeristy |
| Noelle Carlozzi | Indiana Univeristy |
| Shannon A. Johnson | Indiana Univeristy |
| Scott A. Wylie | Indiana Univeristy |
| J. Colin Campbell | Indiana Univeristy |

| Eric J. Peters | Indiana Univeristy |
| --- | --- |
| Petra Theiner-Schumacher | Indiana Univeristy |
| Heather Coates | Indiana Univeristy |
| Sarah Queller | Indiana Univeristy |
| David Caughlin | Indiana Univeristy |
| Terren Green | Indiana Univeristy |
| Shelley Swain | Indiana Univeristy |
| Bethany Ward-Bluhm | Indiana Univeristy |
| Shaun Siegler | Indiana Univeristy |
| **Biomedical Informatics** |  |
| H. Jeremy Bockholt | University of Iowa |
| Roland Zschiegner | University of Iowa |
| Paul Allen | University of Iowa |
| Sudharshan Reddy Bommu | University of Iowa |
| Erin Carney | University of Iowa |
| Robert Connell | University of Iowa |
| Dan Kitzman | University of Iowa |
| Bill McKirgan | University of Iowa |
| Karen Pease | University of Iowa |
| Benjamin Rogers | University of Iowa |
| Adam Scott | University of Iowa |
| Mark Scully | University of Iowa |
| Jim Smth | University of Iowa |
| Austin Suiter | University of Iowa |
| Kent Williams | University of Iowa |
| Shuhua Wu | University of Iowa |
| Ryan Wyse | University of Iowa |
| **Ethics** |  |
| Cheryl Erwin | University of Iowa, U TX, Houston |
| Patricia Backlar | Portland State university |
| Debbie Bury | Chicago-Kent College of Law |
| Jim Calhoun | President, HDSA Georgia |
| Vicki Hunt | Wake Forest University |
| H. Jeremy Bockholt | University of Iowa |
| Thomas Brashers-Krug | University of Iowa |
| Janet K. Williams | University of Iowa |
| Martha Nance | Hennepin Hospital, Minneapolis |
| Jason Evans  Kimberly Quaid | University of Iowa  Indiana University, Indianapolis |
| Steven Hersch | Emory University |
| Lisa Hughes | University of Texas, Houston |
| Carl Leventhal | Indiana University |
| Bernard Lo | University of CA |
| Erik Parens | The Hastings Center |
| Harold Shapiro | Princeton University |
| Aileen Shinaman | University of Rochester |
| Alice Wexler | UCLA |
| Nancy Wexler | Columbia University |
| Jane Paulsen | University of Iowa |

| **Iowa Study Coordination Center:** | |
| --- | --- |
| Elizabeth Penziner | University of Iowa |
| Christine Werling | University of Iowa |
| Karla Anderson | University of Iowa |
| Craig Stout | University of Iowa |
| Kristine Bjork | University of Iowa |
| LeeAnn Davis | University of Iowa |
| Ann Dudler | University of Iowa |
| Jamy Schumacher | University of Iowa |
| Steve Blanchard | University of Iowa |
| Phil Danzer | University of Iowa |
| Kelsey Montross | University of Iowa |
| Brenda Humble | University of Iowa |
| Steve Blanchard | University of Iowa |
| Bryan Ludwig | University of Iowa |
| Anne Leserman | University of Iowa |
| Lynda Sherman | University of Iowa |
| Carissa Nehl, BS | University of Iowa |
| Jane Paulsen | University of Iowa |
| Daniel Fernandez-Baca | University of Iowa |
| Gloria Ellis | University of Iowa |
| Stacie Vik | University of Iowa |
| **MGH DNA Lab:** |  |
| Marcy MacDonald James Gusella | MGH, Harvard University, Boston MGH, Harvard University, Boston |
| **HSG Clinical Trials Coordination Center:** | |
| Keith Bourgeois | University of Rochester |
| Catherine Covert | University of Rochester |
| Susan Daigneault | University of Rochester |
| Karl Kieburtz | University of Rochester |
| Elise Kayson | Elise Kayson, MS, RNC |
| Elaine Julian-Baros | University of Rochester |
| Beverly Olsen | University of Rochester |
| Constance Orme | University of Rochester |
| Tori Ross | University of Rochester |
| Karen Rothenburgh | University of Rochester |
| Joseph Weber | University of Rochester |
| Hongwei Zhao | University of Rochester |
| **IMAGING Working Group:** |  |
| Elizabeth Aylward | University of Washington, Seattle |
| Vince Magnotta | University of Iowa |
| Hans Johnson | University of Iowa |
| Christopher Ross | Johns Hopkins University |
| Stephen Rao | Cleveland Clinic, Ohio |
| Jeremy Bockholt | University of Iowa |
| Peg Nopoulos | University of Iowa |
| Jatin Vaidya | University of Iowa |
| Deborah Harrington | UCSD |

| Andy Feigen | North Shore University |
| --- | --- |
| David Eidelberg | North Shore University |
| Vince Calhoun | MIND Research Network, UNM, GSU |
| Jessica Turner | Georgia State University |
| Michael Miller | Johns Hopkins University |
| CF Westin | Harvard Medical School |
| Guido Gerig | Utah Center for Neuroimaging / NYU |
| **Cognitive Working Group** |  |
| Peter Como | University of Rochester |
| Julie Stout | Indiana University |
| Leigh Beglinger | University of Iowa |
| Susan Bonner | Rhode Island Hospital |
| Noelle Carlozzi | Indiana University/ University of Michigan |
| Gabriel Castillo | UCSD |
| Jennifer Davis | Brown University |
| Michael Diaz | WUSL |
| Ian Dobbins | WUSL |
| Kevin Duff | University of Iowa/University of Utah |
| Greg Elias | Rhode Island Hospital |
| Melanie Faust | Rode Island Hospital |
| Erin Foster | WUSL |
| Carissa Gehl | VAMC Iowa City |
| Nellie Georgiou-Karistianis | University of Melbourne |
| Deborah Harrington | UCSD |
| Tamara Hershey | WUSL |
| Herwig Lange | Air-Rahazentrum |
| Kirsty Metheson | University of Aberdeen |
| Deborah Moore | WUSL |
| Jessica Morison | UCSD |
| KC Rowe | Univrsity of Iowa |
| David Moser | University of Iowa |
| Karen Siedlecki | Fordham University |
| Kate Papp | Brown University |
| Peter Snyder | Brown University |
| Jason Reed | UCSD |
| Megan Smith | University of Iowa |
| Danielle Theriault | University of Iowa |
| Geoff Tremont | Brown University |
| Natalie Valle Guzman | John vanGeest Center for Brain Repair |
| Carol Manning | University of Virginia |
| Randi Jones | Emory Universtiy |
| Holly Westerveld | Brown University |
| **Recruitment Retention Committee:** |  |
| Michelle Fox | University of California, Los Angeles |
| Elise Kayson | University of Rochester |
| Elaine Julian-Baros | University of Rochester |
| Martha Nance | Park Nicollet Clinic |
| Jane Paulsen | University of Iowa |
| Elizabeth Penziner | University of Iowa |
| Kimberly Quaid | Indiana University |

| Amanda Barnes | Johns Hopkins University |
| --- | --- |
| Greg Suter | Hereditary Neurological Disease Centre |
| Andrea Zanko | University of California San Francisco |
| Randi Jones | Emory University |
| Melinda Kavanaugh | Washington University |
| Hillary Lipe | University of Washington |
| Terry Tempkin | University of California Davis |
| Stacy Vik, BA | University of Iowa |
| Rachel Zombor | Graylands, Selby-Lemnos, Perth |
| Sean Thompson | University of Iowa |
| Katrin Barth | University of Ulm |
| Jenny Naji | Cardiff University |
| Jane Griffith | Westmead Hospital |
| Norman Reynolds | Medical College of Wisconsin |
| **Statistics Working Group:** |  |
| Jeffrey Long | University of Iowa |
| Ji-In Kim | University of Iowa |
| Wenjing Lu | University of Iowa |
| James Mills | University of Iowa |
| Blair Harrison | University of Iowa |
| Spencer Lourens | University of Iowa |
| Dawei Liu | University of Iowa |
| Ying Zhang | University of Iowa |
| Kai Wang | University of Iowa |
| Douglas Langbehn | University of Iowa |
| Hongwei Zhao | Texas A&M University |
| David Oakes | Unifersity of Rochester |

| **PREDICT-HD Investigators, Coordinators, Motor Raters, Cognitive Raters,** |
| --- |
| **University of Iowa:** |
| Robert Rodnitzky, Henry Paulson, Ergun Uc, Justin Smock, Jessica Wood, Thomas Brashers- Krug, Jess Fiedorowicz, Eric Epping, Leigh Beglinger, Kevin Duff, Megan Smith, Jane Paulsen, Carissa Nehl Gehl, Karin Hoth, Clare Hey, Elizabeth Penziner, Beth Turner, Lynn Vining, Ania Mikos, Becky Reese, Rachel Conybeare, Sara Vander Heiden, Stacie Vik, Isabella De Soriano, Jessica Schumacher, Erica Wagner, Kimberly Bastic, Michelle Benjamin, Nancy Hale, Katie Hall, Mycah Kimble, Harisa kuburas, Jeremy Hinkel, Andrew Juhl, Stephen Cross, Angel Dominguez, Mackenzie Elbert, Terry Hayes, Jolene Luther, Amanda  Miller, Pat Ryan, Emily Shaw, Kelli Thumma, Owen Wade, Courtney Hobart |
|  |
| **University of Melbourne, Australia:** |
| Edmond Chiu, Phyllis Chua, Joy Preston, Anita Goh, Olga Yastrubetskaya, Samantha Loi, Stephanie Antonopoulos Andrew Gibbs, Phillip Dingjan, Kristy Draper, Chathushka Fonseka, Nellie Georgiou-Karistianis, Christel Lemmon, Liz Ronsisvalle, David Ames, John Lloyd, Angela Komiti, Andrew Gibbs |
|  |
| **University of British Columbia, Vancouver:** |

| Lynn Raymond, Joji Decolongon, Mannie Fan, Allison Coleman, Elisabeth Almqvist, Kimberley Carter, Rachelle Dar Santos, Jordana Hutchinson, David Weir, Blair Leavitt |
| --- |
|  |
| **Johns Hopkins University, Baltimore, MD:** |
| Christopher Ross, Adam Rosenblatt, Lisa Gourley, Arnold Bakker, Robin Miller, Barnett Shpritz, Mark Varvaris, Abhijit Agarwal, Gregory Churchill, Maryjane Ong, Meeia Sherr, Kristine Wajda, Claire Welsh, Nadine Yoritomo, Jason Brandt, Carolin Eschenbach |
|  |
| **Hereditary Neurological Disease Centre, Wichita, KS:** |
| William M. Mallonee, Greg Suter, Judy Addison, David Palmer |
|  |
| **University of Washington & VA Puget Sound Health Care System, Seattle, WA:** |
| Ali Samii, Hillary Lipe, Rebecca Logsdon, Kurt Weaver, Thomas Bird, Rosalynn DeLeon, Emily Freney, Alma Macaraeg |
|  |
| **Emory University School of Medicine, Atlanta, Georgia** |
| Randi Jones, Joan Harrison, Stewart Factor, Cora Bush, Janet Cellar, Carol Ingram, Cathy Wood- Siverio, Timothy Greenamyre, Claudia Testa |
|  |
| **John van Geest Centre for Brain Repair, Cambridge, UK** |
| Roger Barker, Sarah Mason, Emma Smith, Natalie Valle Guzman, Gemma Cummins, Anna Di Pietro, Rachel Swain, Anna Goodman |
|  |
| **Westmead Hospital, Wentworth Ville, Sydney, Australia:** |
| Elizabeth McCusker, Bernadette Bibb, Clement Loy, Catherine Hayes, Kylie Richardson, Jane Griffith, David Gunn, Jillian McMillan |
|  |
| **University of Ulm, Germany:** |
| Bernhard Landwehrmeyer, Michael Orth, Katrin Barth, Carolin Eschenbach, Christine Held, Daniela Schwenk, Daniel Ecker, Anke Niess, Sonja Trautmann, Sigurd Suessmuth, Patrick Weydt |
|  |
| **Indiana University School of Medicine, Indianapolis:** |
| Kimberly Quaid, Melissa Wesson, Kathy Fleming, Jamalynne Stuck, Xabier Beristain, Joanne Wojcieszek |
|  |
| **Centre for Addiction and Mental Health University of Toroto, Ontario, Canada** |
| Mark Guttman, Rosa Ip, Alanna Scheinberg, Deanna Shaddick, Janice Stober, Rustom Sethna,  Catherine Brown, Sheryl Elliott, Zelda Fonariov, Christine Giambattista, Albie Law, Irita Karmalkar, Sandra Russell, Joseph Sebastian, Adam Singer |
|  |
| **University of California, Los Angeles Medical Center, CA** |
| Susan Perlman, Laurie Carr, Russell Carroll, Brian Clemente, Arik Johnson, George Jackson,  Xabier Beristain |
|  |
| **University of California San Francisco:** |
| Michael Geschwind, Joel Kramer, Sharon Sha, Margaret Wetzel, Jonathan Gooblar, Mira Guzijan, Katherine Rose, Gabriela Satris, Joseph Winer, Christina Wyss-Coray |
|  |
| **National Hospital for Neurology and Neurosurgery, London, UK:** |
| Tom Warner, Stefan Kloppel, Charlotte Golding, Thomasin Andrews, Eirini Kalliolia, Elisabeth Rosser, Sarah Tabrizi, Maggie Burrows |
|  |
| **Cardiff University, Cardiff, Wales, UK:** |

| Anne Rosser, Olivia Jane Handley, Jenny Naji, Catherine Johnston, Sarah Hunt, Kathy Price |
| --- |
|  |
| **University of Rochester, Rochester, New York, USA:** |
| Peter Como, Frederick Marshall, Charlyne Hickey, Amy Chesire, Carol Zimmerman, Christina Burton, Mary Wodarski, Timothy Couniham |
|  |
| **Neuroscience Unit, Graylands, Selby-Lemnos & Spec. Care Health Services, Perth, Australia** |
| Peter Panegyres, Rachel Zombor, Brenton Maxwell, Maria Tedesco, Elizabeth Vuletich, Mark Woodman, Joseph Lee |
|  |
| **Washington University – St. Louis, MO, USA** |
| Joel Perlmutter, Stacey Barton, Melinda Kavanaugh, Amy Schmidt, Shineeka Smith, Lori McGee- Minnich |
|  |
| **Clinical Genetics Centre, Aberdeen, Scotland, UK** |
| Sheila Simpsom, Zosia Miedzybrodzka, Jackie Hamilton, Mariella D'Alessandro, Lorna Downie, Gwen Keenan, Kirsty Matheson, Daniella Rae, Alexandra Ure, Vivien Vaughan |
|  |
| **University of Manchester, Manchester, UK:** |
| David Craufurd, Jenny Callaghan, Elizabeth Howard, Judith Bek, Ruth Fullam, Rhona Macleod, Andrea Sollom |
|  |
| **Columbia University Medical Center, New York, NY, USA:** |
| Pietro Mazzoni, Karen Marder, Paula Wasserman, Jennifer Williamson, Paula Leber |
|  |
| **Colorado Neurological Institute, Englewood, Colorado, USA:** |
| Rajeev Kumar, Lauren Seeberger,Carolyn Greenwald, Melissa Holtgrewe, Breanna Nickels, Jay Schneiders, Christopher O'Brien, Colleen Dingmann, Diane Erickson, Carolyn Greenwald, Deborah  Judd, Terri Lee Kasunic, Kristi Malleck, Lisa Mellick, Dawn Miracle, Sherrie Montellano, Christina Reeves, ,Alan Diamond |
|  |
| **University of California – Davis, Sacramento, California, USA** |
| Vicki Wheelock, Terry Tempkin, Kathleen Baynes, Sarah Farias, Lisa Kjer, Nicole Mans, Joseph Marsano, Amanda Martin |
|  |
| **University of Alberta, Edmonton, Alberta, Canada:** |
| Wayne Martin, Sheri Foster, Marguerite Weiler, Oksana Suchowersky, Satwinder Sran, Pamela King |
|  |
| **Cleveland Clinic Foundation, Cleveland, Ohio, USA:** |
| Anwar Ahmed, Jagan Pillai, Stephen Rao, Christine Reece, Justina Baryak, Alexandra Bea, Emily Newman, Alex Bura, Michael Lengen, Lyla Mourany, Juliet Schulz, Janice Zimbelman |
|  |
| **Baylor College of Medicine, Waco, TX, USA** |
| Tetsuo Ashizawa, Joseph Jankovic, Christine Hunter, Norma Cooke, Carrie Martin, Karinna Pacheco, George Ringholtz, Cynthia Studenko, William Ondo, Nicte Mejia, Kevin Dat Nguyen-Vuong, Lynn Ratkos |
|  |
| **University of Calgary, Calgary, AB, Canada:** |
| Oksana Suchowersky, Mary Lou Klimek, Dolen Kirstein, Sharon Lockey, Sarah Furtado, Anne Louise Lafontaine, Dwight Stewart |
|  |

| **Hospital Ramon y Cajal, Madrid, Spain** |
| --- |
| Justo Garcia de Yebenes, Asuncion Martinez-Descales, Marta Fatas, Christine Schwartz, Javier Alegre, Monica Bascunana Garde |
|  |
| **Hennepin County Medical Center, Minneapolis, MN, USA** |
| Martha Nance, Dawn Radtke, Deanna Norberg, David Tupper |
|  |
