# Supplementary material for: Huntington’s disease age at motor onset is modified by the tandem hexamer repeat in TCERG1
Source: NPJ Genom Med. 2022 Sep 5;7:53. doi: 10.1038/s41525-022-00317-w (PMC9445028; doi:10.1038/s41525-022-00317-w)
Supplement: Supplementary file 2 — Reporting Summary [file 41525_2022_317_MOESM2_ESM.pdf]

nature portfolio

Corresponding author(s): Lesley Jones
Last updated by author(s): 24 April 2022

Reporting Summary

Nature Portfolio wishes to improve the reproducibility of the work that we publish. This form provides structure for consistency and transparency in reporting. For further information on Nature Portfolio policies, see our Editorial Policies and the Editorial Policy Checklist.

Statistics

For all statistical analyses, confirm that the following items are present in the figure legend, table legend, main text, or Methods section.

n/a	Confirmed	
☐	☒	The exact sample size (n) for each experimental group/condition, given as a discrete number and unit of measurement
☐	☒	A statement on whether measurements were taken from distinct samples or whether the same sample was measured repeatedly
☐	☒	The statistical test(s) used AND whether they are one- or two-sided<br>*Only common tests should be described solely by name; describe more complex techniques in the Methods section.*
☐	☒	A description of all covariates tested
☐	☒	A description of any assumptions or corrections, such as tests of normality and adjustment for multiple comparisons
☐	☒	A full description of the statistical parameters including central tendency (e.g. means) or other basic estimates (e.g. regression coefficient) AND variation (e.g. standard deviation) or associated estimates of uncertainty (e.g. confidence intervals)
☐	☒	For null hypothesis testing, the test statistic (e.g. F, t, r) with confidence intervals, effect sizes, degrees of freedom and P value noted<br>*Give P values as exact values whenever suitable.*
☒	☐	For Bayesian analysis, information on the choice of priors and Markov chain Monte Carlo settings
☒	☐	For hierarchical and complex designs, identification of the appropriate level for tests and full reporting of outcomes
☐	☒	Estimates of effect sizes (e.g. Cohen's d, Pearson's r), indicating how they were calculated

Our web collection on statistics for biologists contains articles on many of the points above.

Software and code

Policy information about availability of computer code

Data collection	*Provide a description of all commercial, open source and custom code used to collect the data in this study, specifying the version used OR state that no software was used.*
Data analysis	The software performing regression with selection and STR/QTR calling are openly available from https://github.com/LobanovSV at the RegressionWithSelection and UVC repositories, respectively.

For manuscripts utilizing custom algorithms or software that are central to the research but not yet described in published literature, software must be made available to editors and reviewers. We strongly encourage code deposition in a community repository (e.g. GitHub). See the Nature Portfolio guidelines for submitting code & software for further information.

Data

Policy information about availability of data

All manuscripts must include a data availability statement. This statement should provide the following information, where applicable:

- Accession codes, unique identifiers, or web links for publicly available datasets
- A description of any restrictions on data availability
- For clinical datasets or third party data, please ensure that the statement adheres to our policy

The Predict data are available through dbGAP accession number phs000371.v2.p1.
The Registry phenotypic data can be obtained from the Registry legacy database held by the European Huntington's Disease Network (http://www.ehdn.org/). The sequence data are identifying and controlled by our MTA with Registry: we cannot release these data to others. The data are being uploaded to EGA but the accession number is not yet available. We will communicate this as soon as it is known.

Field-specific reporting

Please select the one below that is the best fit for your research. If you are not sure, read the appropriate sections before making your selection.

☒ Life sciences ☐ Behavioural & social sciences ☐ Ecological, evolutionary & environmental sciences

For a reference copy of the document with all sections, see nature.com/documents/nr-reporting-summary-flat.pdf

Life sciences study design

All studies must disclose on these points even when the disclosure is negative.

Sample size	506 participants from the Registry study and 104 participants from the Predict-HD study were exome sequenced and analysed. For the Registry study 250 partcipants at either end of the distribution of residual age at onset (actual age at onset compared with onset predicted by CAG length) from a total of 6086 participants were used (in fact for various reasons we had 507 participants in the final study). This gave 80% power to detect an effect size equivalent to the largest effect size seen in the Gem-HD study reported in Cell in 2015 (ref 7). We augmented this sample with the 104 participants from the Predict-HD study who had both age at onset and exome sequence data available. 468 of the participants from Registry had previously generated SNV data.
Data exclusions	We did not use Predict-HD participants who had no recorded age at onset as they could not be included in our analyses. 1 participant was excluded due to low exome-sequencing read coverage of the TCERG1 STR/QTR (probability of genotyping was below 0.1) and since there was no capillary electrophoresis data for this individual.
Replication	The Predict-HD formally replicates the Registry findings though it is less powerful, and we have chosen to present these as a single analysis. There are no other HD cohorts with sequencing and appropriate clinical data available as far as we are aware.
Randomization	We did not randomise our study. It looked only at participants with HD and used age at onset to analyse the data. The early and late groups were not directly compared in this study but rather the effect of the TCERG1 repeat on age at onset was examined.
Blinding	The analysis used all the subjects available through our original selection process so the participants were not further selected and all the participants were used in the analyses. The TCERG1 STR/QTR calls were performed by an algorithm.

Reporting for specific materials, systems and methods

We require information from authors about some types of materials, experimental systems and methods used in many studies. Here, indicate whether each material, system or method listed is relevant to your study. If you are not sure if a list item applies to your research, read the appropriate section before selecting a response.

Materials & experimental systems

n/a	Involved in the study	
☒	☐	Antibodies
☒	☐	Eukaryotic cell lines
☒	☐	Palaeontology and archaeology
☒	☐	Animals and other organisms
☐	☒	Human research participants
☐	☒	Clinical data
☒	☐	Dual use research of concern

Methods

n/a	Involved in the study	
☒	☐	ChIP-seq
☒	☐	Flow cytometry
☒	☐	MRI-based neuroimaging

Human research participants

Policy information about studies involving human research participants

Population characteristics	The population was recruited from HD gene expansion carriers. The 507 Registry particpants were recruited in the Registry study which was an observational study conducted across Europe with extensive clinical data collected at multiple visits. The Predict-HD study which contributed 232 individuals was also observational. Participants were at risk for Huntington's disease and we only analysed the 104 subjects who had a confirmed age at onset of Huntington's disease.
Recruitment	Participants in Registry and Predict-HD were cohort studies that recruited from multiple clinics in Europe or America/ Australia/Europe respectively. There is potentially some bias in that those who agreed to participate and whose data were collected were more likely to have support to continue in the studies. We do not feel that these characteristics will bias our studies. We used age at motor onset, rated by the clinic physician, which is a more objective measure than onset of cognitive or psychiatric symptoms. This has been used widely in analysing HD age at onset previously.
Ethics oversight	Ethical permission was given for the Registry study (Registry 10/MRE00/19; 10/WSE04/07) and the Predict-HD study. All participants gave informed consent. Investigation of the pseudonimised data from the Registry HD study was approved by

the School of Medicine Ethical Review Board of Cardiff University. Investigation of deidentified Predict-HD subjects was approved by the Institutional Review Board of Partners HealthCare (now Mass General Brigham).

Note that full information on the approval of the study protocol must also be provided in the manuscript.

Clinical data

Policy information about clinical studies

All manuscripts should comply with the ICMJE guidelines for publication of clinical research and a completed CONSORT checklist must be included with all submissions.

Clinical trial registration	*Provide the trial registration number from ClinicalTrials.gov or an equivalent agency.*
Study protocol	*Note where the full trial protocol can be accessed OR if not available, explain why.*
Data collection	*Describe the settings and locales of data collection, noting the time periods of recruitment and data collection.*
Outcomes	*Describe how you pre-defined primary and secondary outcome measures and how you assessed these measures.*
